# Nutritional ketosis as treatment for alcohol withdrawal symptoms in female C57BL/6J mice

**DOI:** 10.1038/s41598-024-55310-3

**Published:** 2024-03-01

**Authors:** Simone Tonetto, Pia Weikop, Morgan Thomsen

**Affiliations:** 1 Laboratory of Neuropsychiatry, Mental Health Center Copenhagen, Copenhagen University Hospital – Mental Health Services CPH, Copenhagen, Denmark; 2 Center for Translational Neuromedicine, University of Copenhagen, Copenhagen, Denmark; 3 Copenhagen Center for Translational Research, Copenhagen University Hospital - Bispebjerg and Frederiksberg Hospital, Copenhagen, Copenhagen, Denmark; 4 Department of Neuroscience, Faculty of Health and Medical Sciences, University of Copenhagen, Copenhagen, Denmark; 5 Laboratory of Neuropsychiatry, Mental Health Center Copenhagen, Copenhagen University Hospital – Mental Health Services CPH, Hovedvejen 17, 1., 2000 Frederiksberg, Denmark

**Keywords:** Addiction, Preclinical research

## Abstract

Upon both acute and prolonged alcohol intake, the brain undergoes a metabolic shift associated with increased acetate metabolism and reduced glucose metabolism, which persists during abstinence, putatively leading to energy depletion in the brain. This study evaluates the efficacy of ketogenic treatments to rescue psychiatric and neurochemical alterations during long-term alcohol withdrawal. Female mice were intermittently exposed to alcohol vapor or air for three weeks, during which mice were introduced to either a ketogenic diet (KD), control diet supplemented with ketone ester (KE) or remained on control diet (CD). Withdrawal symptoms were assessed over a period of four weeks followed by re-exposure using several behavioral and biochemical tests. Alcohol-exposed mice fed CD displayed long-lasting depressive-like symptoms measured by saccharin preference and tail suspension, as well as decreased norepinephrine levels and serotonin turnover in the hippocampus. Both KD and KE rescued anhedonia for up to three weeks of abstinence. KD mice showed higher latency to first immobility in the tail suspension test, as well as lower plasma cholesterol levels. Our findings show promising effects of nutritional ketosis in ameliorating alcohol withdrawal symptoms in mice. KD seemed to better rescue these symptoms compared to KE.

## Introduction

Alcohol withdrawal symptoms are one of the most challenging aspects for the treatment of alcohol use disorder (AUD). During early abstinence these symptoms manifest as intense alcohol craving, negative emotional states, irritability, as well as seizures and delirium tremens in the most severe cases. The subtler long-lasting alcohol withdrawal symptoms, which include dysphoria, anxiety, depression, irritability, and sleep disturbances, contribute to alcohol drinking and relapse. It is estimated that on average 40–60% of abstinent AUD individuals eventually relapse, with incidences as high as 80%^1–3^. Benzodiazepines are the most common and effective therapy for the treatment of alcohol withdrawal symptoms but can lead to addiction on their own^4,5^. Therefore, there is an essential need to better understand the mechanisms leading to AUD and withdrawal to develop more effective treatments.

Brain imaging studies have shown that upon acute and prolonged alcohol intoxication the brain undergoes a metabolic shift associated with reduced glucose metabolism and increased acetate metabolism^6–8^. This metabolic imbalance seems to persist during abstinence, when acetate availability is low, putatively leading to an energy depletion state^8–10^. This is hypothesized to contribute to the genesis and persistency of alcohol withdrawal symptoms. Therefore, it has been hypothesized that providing the brain with an alternative source of energy could ameliorate alcohol withdrawal symptoms. Ketone bodies, i.e., β-hydroxybutyrate (βHB), acetoacetate and acetone, are produced from fatty acids when glucose levels are low, e.g., during starvation or prolonged exercise. Ketone bodies, like acetate, are transported via monocarboxylate transporters to target cells, fueling the tricarboxylic acid cycle as acetyl-CoA, and thus serving as auxiliary energy supply^11–13^. Nutritional ketosis is a physiological state characterized by elevated ketone bodies levels (> 0.5 mM). It can be achieved via fasting, via a ketogenic diet (KD; very high fat, moderate protein, very low carbohydrate) or via exogenous ketone supplementation, such as D-βHB ketone salts or ketone esters (KE).

It has been described that a KD or ketone salt supplementation alter brain metabolism and that the partial substitution of ketone bodies for glucose as a fuel decreased glucose metabolism and increased acetate metabolism^14,15^. Recent preclinical and clinical studies have shown that nutritional ketosis states ameliorate acute alcohol withdrawal symptoms in rodents and decrease the need for benzodiazepine intake during detoxification in AUD individuals^16–18^. Specifically, it has been shown that a KD during alcohol exposure reduced early withdrawal signs in male rats^16^. It has also been shown that both KD and KE administered throughout alcohol exposure reduced early somatic alcohol withdrawal symptoms and anxiety-like behaviors in male mice, showing that ketone bodies are sufficient to reduce withdrawal symptoms (e.g., reduced carbohydrates are not necessary)^17^. Further, KE but not KD reduced the early somatic signs when administered at the start of abstinence^17^. This could be explained by the faster elevation in ketone bodies achieved by KE than KD (minutes vs. days)^17,19^ and shows that these symptoms can also be rescued when the diet is implemented at withdrawal onset (rather than during alcohol exposure), as may be implemented in a clinical setting.

Despite these promising effects of nutritional ketosis as adjuvant therapy for alcohol withdrawal, it is unclear whether ketosis can attenuate lasting withdrawal symptoms, which are linked to relapse. Moreover, despite an increase in alcohol consumption rates, particularly among young women, data in female subjects are lacking and they are underrepresented in clinical trials^20,21^. In this study, we investigated for the first time the effects of nutritional ketosis on long-lasting alcohol withdrawal symptoms in female mice. To induce nutritional ketosis, we used both a KD and an exogenous KE (ketone monoester (R)-3-hydroxybutyl (R)-3-hydroxybutyrate), since adherence to KD could be challenging and thus KE supplementation would be easier to implement. Further, comparing KD and KE effects helps clarify mechanisms of action of nutritional ketosis, i.e., evaluate the hypothesis that beneficial effects are due to ketone body utilization alone. Based on our previous feasibility study, we selected the C57BL/6JRj mouse strain and performed a battery of behavioral tests to assess alcohol self-administration, anhedonia, hyperalgesia, anxiety-like and depressive-like disturbances as well as ultrasonic vocalizations (USVs). The test diets may affect alcohol absorption or metabolism^17^, potentially leading to different blood alcohol levels that would complicate the interpretation of any effects on withdrawal symptom severity. Therefore, we used alcohol vapor exposure and an alcohol dehydrogenase inhibitor to produce comparable blood alcohol levels across groups. KD can produce a wide range of effects^22–25^, and it remains to be determined whether mechanism other than energy metabolism contribute to withdrawal amelioration. To further clarify the mechanisms underlying the behavioral effects, we assessed biochemical effects of KD and KE on neurotransmitters and neuroinflammatory markers, since KD has been shown to produce anti-inflammatory effects^26,27^.

## Material and methods

### Subjects

72 female C57BL/6JRj mice (Janvier Labs, Le Genest Saint Isle, France) were acquired at six weeks of age and acclimated one week to the facilities before testing. Mice were group-housed four per cage (not necessarily littermates) with hiding devices, nesting material, and wooden chewing blocks as enrichment, under a reversed 12 h light–dark cycle (light on 19:00–07:00). Tap water was available ad libitum throughout the study, and standard rodent chow (Altromin 1310, Brogaarden, Denmark) was available ad libitum until switching to experimental diets. Experimental diets were provided as described below. Before the beginning of experiments, mice were anesthetized briefly using sevoflurane to insert an RFID microchip (Uno Micro-ID/12 transponders, UNO-lifescience, Zevenaar, NL). At the end of all experiments, animals were euthanized by decapitation followed by immediate blood and brain samples collection for further analyses. Procedures were approved by the Animal Experiments Inspectorate under the Danish Ministry of Food, Agriculture, and Fisheries in accordance with the EU directive 2010/63/EU. The study was carried out in compliance with the ARRIVE guidelines.

### Chronic intermittent alcohol vapor exposure

Chronic Intermittent alcohol vapor exposure (CIE) was obtained by vaporizing ethanol (96%), which was then mixed with fresh air, and delivered to inhalation chambers at a rate of 11 L/min, resulting in vapor concentrations of 6–10 mg/L, using custom chambers built after Wang et al.^28^. CIE protocol consisted of a 4-day ON and 3-day OFF cycle of alcohol vapor. ON days were 16 h alcohol vapor (from 19:00 to 11:00 on the following day) followed by 8 h air withdrawal. CIE mice were injected with the alcohol dehydrogenase inhibitor pyrazole (1 mmol/kg, i.p. in a volume of 10 mL/kg body weight) and a loading dose of alcohol (1.6 g/kg) prior placement into the vapor chambers to maintain a high and stable level of intoxication during alcohol exposure^29^. Control air-exposed mice were subjected to the same chamber conditions with air instead of alcohol vapor and were administered pyrazole in saline before being placed into the control chambers. The housing conditions in the inhalation chambers were similar to those in the colony room (bedding, enrichment, food and water, etc.).

### Experimental diets

Isocaloric liquid diets were developed in house, as previously described^17^, and were the following: control diet (CD); ketogenic diet (KD), with 90% of the calories provided by fat; control diet supplemented with KE, with 30% of the calories provided by the KE itself ((R)-3-hydroxybutyl (R)-3-hydroxybutyrate, unflavored, ΔG Tactical Ketones, TdeltaS Global, Orlando, FL, USA) replacing 3% calories from fat, 17% from carbohydrates and 10% from proteins. CD consisted of the following ingredients (for 1 L solution): 22 g KetoCal (a nutritionally complete, ketogenic formula with a 4:1 fat:(carbohydrate + protein) ratio; Nutricia, Amsterdam, NL); 486.3 mL black currant flavor Nutridrink juice style (a carbohydrate and whey protein-based, vitamin-mineral enriched nutrition drink; Nutricia); 31.7 g casein protein powder; 6.5 g fibers (cellulose, xantham gum, psyllium husk); 453.5 mL filtered tap water. KD consisted of the following ingredients (for 1 L solution): 144.5 g KetoCal; 2.8 g fibers (cellulose, xantham gum, psyllium husk); 853 mL filtered tap water. KE consisted of the following ingredients (for 1 L solution): 18.9 g KetoCal; 361.4 mL black currant flavor Nutridrink juice style; 7.5 g casein protein powder; 6.7 g fibers (cellulose, xantham gum, psyllium husk); 549 mL filtered tap water; 56.6 ml (60.3 g) of ketone monoester.

**Figure 1:**
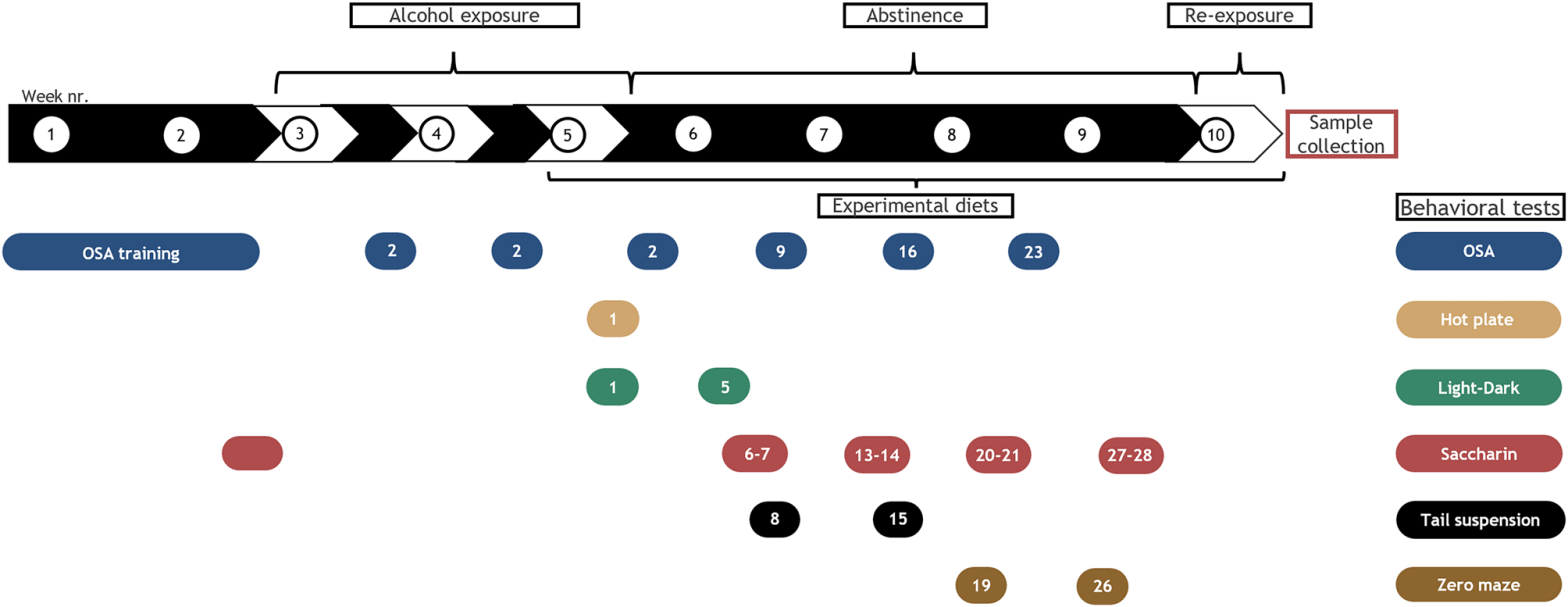
Timeline for alcohol exposure, abstinence, and behavioral assays. Alcohol exposure is highlighted in white and withdrawal periods in black. Behavioral tests are indicated by the colored bubbles, with the testing day specified inside, counted as days of forced abstinence within each specific withdrawal period. Blood for BAL measurement was collected at the end of each alcohol exposure cycle, i.e., at the end of each white arrow. Oral self-administration (OSA).

Experimental diets were administered starting from the third alcohol exposure cycle (Fig. 1). For feasibility and internal replication, mice were tested in three consecutive cohorts balanced for treatment (i.e., randomized block design) with each group represented in all cohorts (n = 12 per group). Mice were randomly assigned to one of 6 groups:

- alcohol-exposed and CD (CIE-CD)
- alcohol-exposed and KD (CIE-KD)
- alcohol-exposed and KE (CIE-KE)
- air-exposed and CD (air-CD)
- air-exposed and KD (air-KD)
- air-exposed and KE (air-KE)

The experimenter was blinded to experimental group for all behavioral tests and sample analyses.

### Operant oral alcohol self-administration (OSA)

Mice were trained to self-administer a 20% alcohol solution in operant-conditioning chambers (ENV-307A, Med Associates Inc, St Albans, VT, USA) prior to CIE, as previously described^30^. Experimental sessions were performed at the first day of each withdrawal window and then once a week in the long-term abstinence.

### Nociception

The hot-plate test, a supraspinal thermal pain assay, was used to measure pain sensitivity 4 h after the last alcohol vapor exposure. Each mouse was placed on a horizontal metal surface (Hot Plate Analgesia Meter, Harvard Apparatus Limited, Holliston, MA, USA) preheated to 52 °C and was confined to the plate with a tall Plexiglas cylinder. The testing room was set to low illumination (≈ 25 lx; Fisherbrand Traceable dual-range light meter, Traceable, Webster, TX, USA). The latency to first licking of hind paws or jumping was recorded. In the absence of a paw licking or jumping response, a 60 s cut-off was used to prevent tissue damage.

### Anxiety-like behaviors

Acute anxiety-like symptoms were assessed by the light–dark transition test on withdrawal days 1 and 5, and was conducted in activity chambers fitted with beam-break movement detection systems (OFA-510, Med Associates), as previously described^17^. The light side had low illumination (≈ 40 lx) not anxiogenic alone^31^, allowing detection of anxiogenic-like effects of alcohol withdrawal^32,33^. The voluntary time spent in the lit area, i.e., the total time spent in the lit area minus the latency to enter the dark area, the total number of crossings between light and dark area and the total distance travelled were analyzed.

Long-lasting anxiety-like symptoms were assessed by the elevated zero maze on withdrawal days 19 and 26, as previously described^34^. The behavioral room was set to medium illumination (≈ 300 lx). The parameters were manually scored in real time and included the time spent on the open areas, the number of entries into the open areas and the number of stretched-attend postures (SAPs).

### Depressive-like behaviors

Depressive-like symptoms were assessed using the tail suspension test on withdrawal days 8 and 15^35^. Depressive-like behavior is exhibited by a decrease in the latency to the first episode of immobility and an increase in immobility time. Mice were suspended upside down by taping their tails to a metal bar. The mice were positioned to prevent them from climbing or reaching nearby surfaces and from seeing each other. A hollow cylinder 4 cm long was placed around the tail to prevent them from climbing it. The behavioral room was set to low illumination (≈ 25 lx). The test lasted 6 min and was recorded for later scoring. The latency to the first immobility bout and the duration of immobility were manually scored for the total duration of the test.

The saccharin preference test was also used to assess anhedonia on withdrawal days 6–7, 13–14, 20–21, and 27–28. We used saccharin rather than sucrose to avoid potential confounders related to caloric content or diet composition. A decrease in sucrose or saccharin intake and preference over water is taken as a putative sign of anhedonia in rodents^36^. Mice were tested in a fluid-intake automated monitoring system (HM-2 system, MBRose, Faaborg, Denmark). The system was in the colony room and consisted of cages very similar to the animals’ regular home cages, with two 11 cm long channels allowing only one mouse at a time access to each bottle. Each channel was equipped with two pairs of microchip readers (one pair at each end) and continuously recorded fluid intake for individual mice^37^. The system was equipped with a scale under each bottle, allowing precise recording of any fluid intake only when a mouse was present in the channel and controlling for spillage by recording any weight added to a drip receptacle under the bottles. Mice were habituated to the system for 48 h prior to alcohol exposure and given a free choice between two bottles, one with 0.1% (w/v) saccharin solution and another with tap water, regularly changing the bottles’ positions to avoid side preferences. The 48-h test sessions used the same conditions. Saccharin preference was calculated as saccharin-solution intake divided by total volume of liquids consumed. No preference was considered when saccharin preference was < 65%.

### USVs

It has been suggested that rodent USVs reflect the stressful nature of drug withdrawal and the anticipatory positive affect of rewarding stimuli, including alcohol^38^. Ultrasonic calls were recorded with an Avisoft UltraSoundGate 416 Hb recording interface (Model CM16-CMPA,Avisoft Bioacoustics, Glienicke/Nordbahn, Germany) during the tail suspension test, and in the first hour of two progressive ratio (PR) OSA sessions. USV calls were recorded and analyzed as previously described^30^.

### Blood measures

At multiple timepoints, blood samples were collected on ice, centrifuged at 3000 rpm for 10 min and plasma was stored at − 20°. Blood alcohol levels (BALs) were determined at the end of each alcohol exposure cycle using an Analox GL5 or GL6 analyzer (Analox Instruments, Stokesley, UK). Blood glucose levels were assessed using strips and FreeStyle Precision Neo analyzer (Abbott), Fridays between 11:00 and 15:00, after the behavioral test if any occurred on that day. Blood βHB levels were similarly assessed using strips and FreeStyle Precision Neo analyzer (Abbott), typically Tuesday and Fridays between 11:00 and 15:00 (in such cases, sampling was delayed until after behavioral testing). Animals were sacrificed 2–4 h after the last cycle of alcohol exposure, and trunk blood was collected in EDTA-coated tubes on ice, centrifuged at 3000 rpm for 10 min and plasma was stored at − 20°. Cholesterol and triglyceride levels were measured from the terminal plasma using a GL5 analyzer.

### High-performance liquid chromatography (HPLC)

Hippocampus, striatum, and cerebellum were dissected from one hemisphere under microscope by an experienced experimenter between 2 and 4 h after the last cycle of alcohol exposure, weighed and directly snap frozen at − 80°C. Hippocampus and striatum samples were each homogenized in 250 µL 0.1 M perchloric acid using an immersion hand disperser, Polytron PT 1200 E (Kinematica Inc., Keyland Court Bohemia, NY, USA). Cerebellum samples were homogenized with the same method in 500 µL milliQ water. Samples were then centrifuged at 14,000 rpm at 4 °C for 20 min and then supernatant collected using a 0.22 µm filter (Avantec, 13CP020AS).

The concentration of norepinephrine (NE), 3,4-dihydroxyphenylacetic acid (DOPAC), dopamine (DA), 5-hydroxyindoleacetic acid (5-HIAA), homovanillic Acid (HVA), and serotonin (5-HT) in both striatum and hippocampus were determined by HPLC with electrochemical detection, as previously described^30^.The concentration of cations, i.e. sodium (Na^+^), magnesium (Mg^++^), potassium (K^+^), and calcium (Ca^++^) in the cerebellum were determined by high-performance ion chromatography (IC, Dionex Aquion 1100, Thermo Fisher Scientific) as previously described^30^.

### Neuroinflammation assay

Brain samples from one hemisphere, except hippocampus, striatum, and cerebellum, were dissected 2–4 h after the last cycle of alcohol exposure, weighed, and directly snap frozen at − 80 °C. Brain sample weights were around 100–130 mg and were homogenized by MagNa Lyser Instrument (F. Hoffmann-La Roche Ltd, Basel, Switzerland). Brain samples were transferred to MagNa Lyser tubes containing 750 µL of tissue extraction reagent II (FNN0081, Thermo Fisher Scientific, Waltham, MA, USA) and 2 × protease inhibitor cocktail (P8340, Merck KGaA, Darmstadt, Germany). The homogenization protocol consisted of two rounds of 25 s at 6000 rpm, with 90 s break on ice. Samples were then spun at 16,000 rcf at 4 °C for 1 min to remove some of the foam, transferred to Eppendorf tubes and then centrifuged at 16,000 rcf at 4 °C for 20 min. Supernatant was aliquoted and frozen at – 80 °C. The total protein concentration was measured by Bradford assay (Merck KGaA, Darmstadt, Germany). The tumor necrosis factor (TNF)-α levels were detected by ELISA (# 88-7324-86, Thermo Fisher Scientific) following manufacturer instructions. Brain samples were loaded 1:4. Final concentrations were adjusted to the brain sample weights and protein concentrations.

### Principal component analysis (PCA)

To deconvolute multidimensional phenotypes associated with each treatment and diet, a PCA analysis was performed for each diet ‘pair’ (CIE and air) with all variables that showed an alcohol effect, i.e., a significant difference between CIE-CD and air-CD mice. We used the number of light/dark crossings on withdrawal day 1, the average of the first 3 saccharin preference tests, the average of the two tail suspension latencies, the NE levels from hippocampus and striatum, and the Mg^++^ and Na^+^ levels. Data were standardized and principal components were selected when eigenvalues were > 1. Data were analyzed using Prism (Version 10, GraphPad, San Diego, CA, USA).

### Statistical analyses

Group sizes were selected based on pilot studies^30^. Data were analyzed with alcohol exposure and diet as between-subject factors and cohort as a blocking factor, followed by planned pairwise comparisons with all CIE groups plus the air-CD mice compared to the CIE-CD mice, and air-exposed groups compared to the air-CD mice. In addition, for the ketone levels, KD and KE groups were compared to each other. Bodyweight, BALs, ketone and glucose levels were each analyzed by mixed model repeated measures (MMRM) with time as repeated factor, alcohol exposure (except for BALs) and diet as between-subject factor, followed by planned pairwise comparisons for each withdrawal day, adjusted for multiple comparisons using the Hochberg approach. For the OSA data, a linear curve was fitted to each animal, followed by one-way analysis of co-variance (ANCOVA) on the intercept and slope parameters with baseline as the covariate. Saccharin preference and consumption, light–dark, tail suspension, elevated zero maze, cholesterol, and triglycerides data were analyzed by two-way analysis of variance (ANOVA) with alcohol and diet as between-subject factors, followed by planned pairwise comparisons using the Hochberg approach. Latencies to events were analyzed by cox proportional hazards regression model. HPLC data were analyzed by two-way ANOVA, with alcohol and diet as between-subject factors, followed by planned pairwise comparisons using the Hochberg approach. Neuroinflammation assay was run in triplicates and the median analyzed by two-way ANOVA. HPLC data, ketone and glucose levels were log(10) transformed to satisfy the parametric analysis assumptions. HPLC datapoints were considered as outliers and excluded from analyses when standard deviation differed more than four times from the group mean (three datapoints in total). Statistical analyses were performed using InVivoStat (Version 4.7.0) and R (version 3.6.3). Graphs were plotted using Prism (Version 10, GraphPad). Bodyweight, ketone and glucose levels, BALs, OSA and saccharin data are shown as group means ± SEMs. All other data are presented as individual subjects, either with violin plots representing group means, distribution, and density or as survival curves. Differences were reported as statistically significant by convention for *p* values < 0.05.

### Ethics declaration

The animal study was reviewed and approved by Animal Experiments Inspectorate under the Danish Ministry of Food, Agriculture, and Fisheries.

## Results

### Both KD and KE induced nutritional ketosis and reduced blood glucose levels at specific timepoints, and KD also reduced plasma cholesterol levels

**Figure 2:**
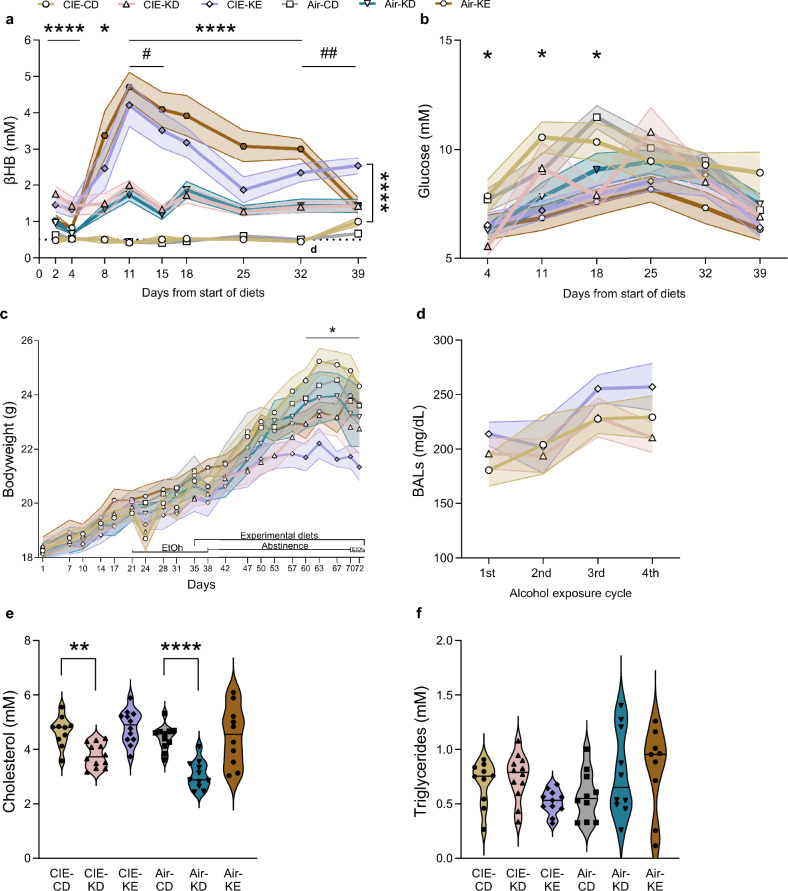
Both KD and KE induced nutritional ketosis and reduced blood glucose levels at specific timepoints, and KD lowered cholesterol levels independently of alcohol exposure. Blood ketone levels (**a**) and glucose levels (**b**) were measured on multiple days. Bodyweight in grams during the whole experiment (**c**). BALs for the alcohol-exposed mice (**d**). Plasma total cholesterol levels in CIE (**e**) and plasma triglycerides levels (**f**). Data are shown as group means, with the shaded area representing ± SEMs and the filled symbols representing a significant effect (**a-d**). Data are shown as individual subjects, horizontal line in violin plots represents group means (**e–f**). n = 10–12 (5–12 for panel **d**). *p < 0.05, **p < 0.01, ***p < 0.001 and ****p < 0.0001. # and ##*p* < 0.05 and *p* < 0.01 (KD vs. KE). Blood alcohol levels (BALs).

Both KD and KE achieved ketosis (i.e., βHB > 0.5 mM), and both also reduced glucose levels (Fig. 2a,b). MMRM analysis revealed a significant overall effect of diet and time (F_2,63_ = 456.1, *p* < 0.0001 and F_8,512_ = 15.8, *p* < 0.0001 respectively), alcohol-time interaction (F_8,512_ = 6.2, *p* < 0.0001) and alcohol-diet-time interaction (F_16,512_ = 2.9, *p* = 0.0001) but not of alcohol (F_1,63_ = 0.9, *p* = 0.4) for blood ketone levels (Fig. 2a). Post hoc analysis revealed that both CIE-KD and CIE-KE mice showed significantly higher ketone levels compared to CIE-CD on withdrawal days 2–4 and 11–32 (*p* < 0.0001), and 8 (*p* = 0.01 and *p* = 0.0002). Only CIE-KE mice showed significant higher ketone levels on withdrawal day 39 (*p* < 0.0001), potentially due to an increase in ketone levels in CIE-CD mice rather than a decrease in CIE-KD mice. In addition, CIE-KE mice had higher ketone levels compared to CIE-KD mice on withdrawal days 11, 15, 32 and 39 (*p* = 0.02, *p* = 0.0004, *p* = 0.01, and *p* = 0.009 respectively), with a trend on day 18 (*p* = 0.066). Similarly, post hoc analysis revealed that both air-KD and air-KE mice showed significantly higher ketone levels compared to air-CD on withdrawal days 8 (*p* = 0.02 and *p* < 0.0001), 11–32 (*p* < 0.0001), and 39 (*p* = 0.0003 and *p* = 0.001). In addition, air-KE mice had higher ketone levels compared to air-KD mice on withdrawal days 11–32 (*p* < 0.0001, *p* < 0.0001, *p* = 0.03, *p* = 0.01, and *p* < 0.0001).

MMRM analysis revealed a significant overall effect of diet and time (F_2,63_ = 23.7, *p* < 0.0001 and F_5,316_ = 19.7, *p* < 0.0001 respectively) but not of alcohol (F_1,63_ = 0.3, *p* = 0.6), alcohol-time interaction (F_5,316_ = 1.3, *p* = 0.3) or alcohol-diet-time interaction (F_10,316_ = 1, *p* = 0.5) for plasma glucose levels (Fig. 2b). Post hoc analysis showed that CIE-KD mice displayed lower plasma glucose levels on withdrawal day 4 (*p* = 0.04) and CIE-KE mice on withdrawal day 11 (*p* = 0.01) compared to CIE-CD. Air-KE mice displayed lower plasma glucose levels compared to air-CD on withdrawal day 18 (*p* = 0.002).

KE reduced bodyweight gains in the later weeks following alcohol exposure (Fig. 2c). MMRM analysis revealed a significant overall effect of time (F_20,1252_ = 282.6, *p* < 0.0001), alcohol-diet-time interaction (F_40,1252_ = 1.6, *p* = 0.009) but not of alcohol and alcohol-time interaction (F_1,63_ = 2.2, *p* = 0.1 and F_20,1252_ = 1.5, *p* = 0.07 respectively) for weight changes (Fig. 2c). Post hoc analysis revealed that CIE-KE mice showed significantly lower weight compared to CIE-CD on days 60–72 (all *p* ≤ 0.015).

BALs averaged between 184.7 and 255.4 mg/dL across all groups and timepoints (Fig. 2d). MMRM analysis revealed a significant overall effect of diet (F_2,28_ = 3.8, *p* = 0.04), time (F_3,61_ = 3.6, *p* = 0.02), but not of diet-time interaction (F_6,61_ = 0.5, *p* = 0.8). There were no statistically significant post hoc differences (all *p* > 0.6).

KD reduced plasma cholesterol, but not triglycerides, independently of alcohol exposure (Fig. 2e,f). Two-way ANOVA revealed a significant overall effect of alcohol and diet for plasma cholesterol levels (F_1,58_ = 8.6, *p* = 0.005 and F_2,58_ = 26.9, *p* < 0.0001 respectively), but not of alcohol-diet interaction (F_2,58_ = 0.8, *p* = 0.5) (Fig. 2e). Post hoc analysis indicated that CIE-KD and air-KD mice had significantly lower plasma cholesterol levels compared to CIE-CD and air-CD mice (*p* = 0.005 and *p* < 0.0001 respectively). There was no significant overall effect of alcohol or diet (F1,55 = 0.2, *p* = 0.6, F2,55 = 0.8, *p* = 0.4 respectively) or evidence that the effect of alcohol varied with diet (F2,55 = 1.5, *p* = 0.2) in plasma triglycerides levels.

### Nutritional ketosis mitigated long-lasting depressive-like symptoms during alcohol withdrawal

**Figure 3:**
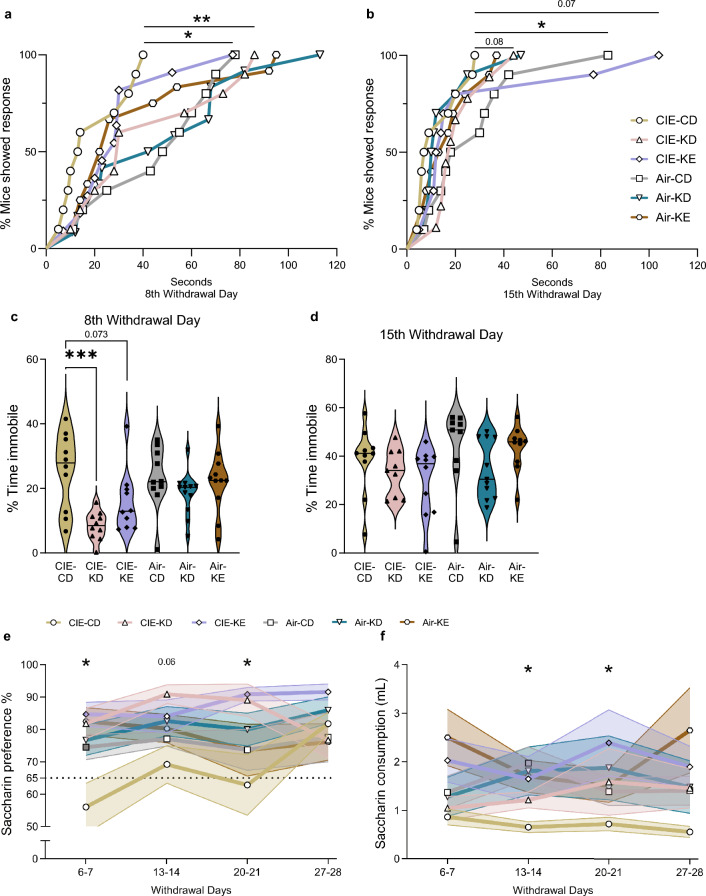
Nutritional ketosis ameliorated depressive-like disturbances induced by alcohol exposure. The latencies in seconds to first immobility (**a**, **b**) and the % of time spent immobile (**c**, **d**) were assessed with the tail suspension test on withdrawal days 8 and 15. The % of saccharin preference (**e**) and the saccharin solution intake (**f**) were assessed on multiple withdrawal days. Latencies are shown as the % of mice showing a response as a function of time (**a**, **b**) and the filled symbols represent a significant effect. Data are shown as individual subjects, horizontal line in violin plots represents group means (**c**, **d**). Data are shown as group means, with the shaded area representing ± SEMs and the filled symbols representing a significant effect (**e**, **f**). Note the truncated ordinate in panel **e**. n = 8–12. *p < 0.05, **p < 0.01 and ***p < 0.001.

CIE-CD displayed long-lasting depressive-like symptoms, assessed by tail suspension test, which were ameliorated by KD (Fig. 3a-d). The two timepoints were analyzed separately, since it is known that mice can habituate^35^. Cox proportional hazards regression analyses revealed that CIE-CD mice had significantly shorter latencies to first immobility compared to air-control mice on both the 8th and 15th withdrawal days (*p* = 0.008 and *p* = 0.03 respectively; Fig. 3a,b), interpreted as more depressive-like behavior. CIE-KD mice had a higher latency to first immobility compared to CIE-CD mice (*p* = 0.03) on withdrawal day 8 (Fig. 3a). On withdrawal day 15, both CIE-KD and CIE-KE showed a trend (*p* = 0.08 and *p* = 0.07 vs. CIE-CD) (Fig. 3b). Two-way ANOVA similarly revealed a significant overall effect of alcohol and diet (F_1,55_ = 4.2, *p* = 0.045 and F_2,55_ = 7.6, *p* = 0.001 respectively) and a trend for alcohol-diet interaction (F_2,55_ = 2.6, *p* = 0.085) for time spent immobile on withdrawal day 8 (Fig. 3c). Post hoc analysis indicated that CIE-KD and KE mice showed lower time spent immobile compared to CIE-CD (*p* = 0.0004 and *p* = 0.07 respectively). However, air-CD mice did not differ significantly from CIE-CD mice. There was a significant overall effect of alcohol (F_1,51_ = 4.2, *p* = 0.045) for time spent immobile on withdrawal day 15, but no post hoc differences (all *p* > 0.4) (Fig. 3d). There were no other statistically significant differences in any parameter in the air-control mice at either timepoint (all *p* > 0.2).

To further investigate depressive-like symptoms, we performed the saccharin preference test once a week for four weeks during alcohol withdrawal. CIE-CD displayed long-lasting decreases in saccharin preference and intake relative to air-control (Fig. 3e,f), interpreted as anhedonia. This symptom was significantly ameliorated by both KD and KE. The four timepoints were analyzed separately, since it is known that mice can habituate and because it was uncertain how long alcohol withdrawal symptoms would last^36,39^. CIE-KD mice from the 3rd cohort were excluded due to an equipment malfunction. Two-way ANOVA revealed significant overall effects of diet for saccharin preference on withdrawal days 6–7 (F_2,59_ = 9.3, *p* = 0.0003), 13–14 (F_2,56_ = 3.8, *p* = 0.03), 20–21 (F_2,59_ = 5.3, *p* = 0.007), but not on days 27–28 (F_2,56_ = 0.2, *p* = 0.8) and of alcohol-diet interaction on withdrawal days 6–7 (F_2,59_ = 4.2, *p* = 0.02), 27–28 (F_2,56_ = 3.3, *p* = 0.04), a trend on days 20–21 (F_2,59_ = 2.6, *p* = 0.08), but not on days 13–14 (F_2,56_ = 1.3, *p* = 0.3), but no overall alcohol effect at any timepoint (Fig. 3e). Post hoc analyses indicated that CIE-CD mice had lower and no (defined as preference < 65%) saccharin preference compared to air-CD on withdrawal days 6–7 (*p* = 0.01) and no saccharin preference on withdrawal days 20–21. CIE-KD and KE mice displayed higher saccharin preference compared to CIE-CD on withdrawal days 6–7 and 20–21 (*p* = 0.002 and *p* = 0.0001; *p* = 0.01 and *p* = 0.005 respectively) and CIE-KD also displayed a trend on withdrawal days 13–14 (*p* = 0.06). Similarly, two-way ANOVA revealed significant overall effects of alcohol for saccharin intake only on withdrawal days 13–14 (F_1,56_ = 5.1, *p* = 0.03), of diet on withdrawal days 6–7 (F_2,59_ = 5.9, *p* = 0.005), 20–21 (F_2,59_ = 3.1, *p* = 0.05), and a trend on days 27–28 (F_2,56_ = 2.9, *p* = 0.06), and a trend of alcohol-diet interaction on withdrawal days 13–14 (F_2,56_ = 2.6, *p* = 0.08) (Fig. 3f). Post hoc analyses indicated that CIE-CD mice showed lower saccharin consumption compared to air-CD on withdrawal days 13–14 (*p* = 0.02). CIE-KE mice displayed higher saccharin consumption on withdrawal days 20–21 (*p* = 0.03) and a trend on days 13–14 and 27–28 (*p* = 0.07 *p* = 0.08) compared to CIE-CD. There were no other statistically significant differences in the saccharin preference or consumption in the air-control mice at any timepoint (all *p* > 0.15).

### KD had modest effects on alcohol-induced anxiety-like symptoms during alcohol withdrawal

**Figure 4:**
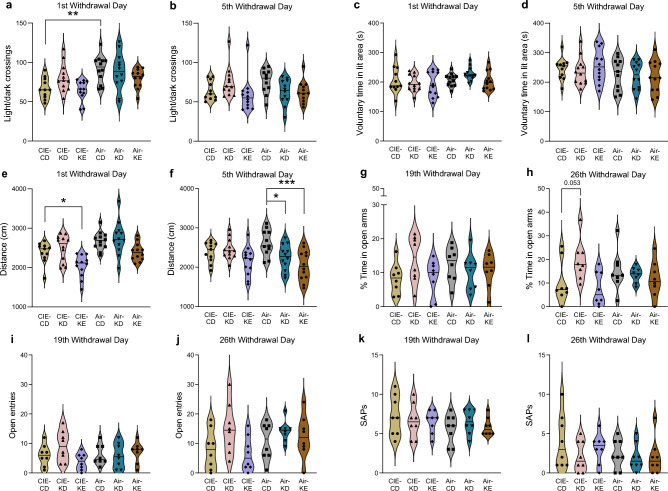
KD had modest effects on anxiogenic-like behaviors during alcohol withdrawal. The number of light/dark crossing (**a**, **b**), the % of time spent voluntarily in the lit area (**c**, **d**), and the distance travelled (**e**, **f**) were assessed with the light/dark test on withdrawal days 1 and 5. The % of time spent in the open arms (**g**, **h**), the number of entries into open arms (**i**, **j**), and the number of stretched-attend postures (SAPs) (**k**, **l**) were assessed with the elevated zero maze on withdrawal days 19 and 26. Data are shown as individual subjects, horizontal line in violin plots represents group means. Note the truncated ordinate in panel **g**. n = 8–12. *p < 0.05, **p < 0.01 and ***p < 0.001.

CIE-CD mice displayed acute anxiety-like symptoms measured in the light–dark transition test, which tended to be rescued by KD (Fig. 4a-f). The two timepoints were analyzed separately, since it is known that mice can habituate to anxiogenic effects in the test. For withdrawal day 1, two-way ANOVA revealed a significant overall alcohol and diet effect for number of light/dark crossings (F_1,63_ = 16.1, *p* = 0.0002 and F_2,63_ = 3.3, *p* = 0.04) and distance travelled (F_1,63_ = 16.9, *p* = 0.0001 and F_2,63_ = 11.3, *p* < 0.0001) and a trend for alcohol effect for voluntary time spent in the light area (F_1,63_ = 3.5, *p* = 0.06) (Fig. 4a,c,e). Post hoc analyses indicated that CIE-CD mice had significantly lower light/dark crossings compared to air-CD mice (*p* = 0.004) and a trend for lower distance travelled (*p* = 0.09). In addition, CIE-KE mice travelled less than CIE-CD mice (*p* = 0.046) and air-KE tended to travel less than air-CD (*p* = 0.085). For withdrawal day 5, there was a small overall effect of alcohol for voluntary time spent in the light area (F_1,62_ = 4.1, *p* = 0.047), diet and alcohol-diet interaction for distance travelled (F_2,62_ = 9, *p* = 0.0004 and F_2,62_ = 2.9, *p* = 0.06 respectively) and alcohol-diet interaction for the number of light/dark crossings (F_2,62_ = 3.3, *p* = 0.045) (Fig. 4b,d,f). Post hoc analyses indicated that both air-KD and air-KE ambulated significantly less on withdrawal 5 compared to air-CD (*p* = 0.04 and *p* = 0.0004 respectively). There were no other statistically significant differences in the number of light/dark crossing and in the voluntary time spent in the light area in the air-control mice at either timepoint (all *p* > 0.2).

CIE-CD mice did not display long-lasting anxiety-like symptoms as measured in the zero maze (Fig. 4g-l). Data from the 2nd and 3rd cohorts were included in the analyses, while the 1^st^ cohort was excluded due to different light conditions. The two timepoints were analyzed separately, since it is known that mice can habituate. On withdrawal day 19, two-way ANOVA revealed no main effects or interactions for the time spent in the open arms or the number of SAPs, but there was a trend for an alcohol-diet interaction for the number of open entries (F_2,41_ = 2.9, *p* = 0.07) (Fig. 4g,i,k). On withdrawal day 26, two-way ANOVA revealed a significant overall effect of diet for the number of open arm entries and for the time spent in the open arms (F_2,41_ = 4.5, *p* = 0.02 and F_2,41_ = 3.95, *p* = 0.03 respectively) as well as a trend for a significant alcohol-diet interaction for the latter (F_2,41_ = 3.1, *p* = 0.06), but no effects for the number of SAPs (Fig. 4h,j,l). Post hoc analyses revealed that CIE-KD mice displayed trends towards more time spent in the open arms compared to CIE-CD mice (*p* = 0.053) and more entries into the open arms (*p* = 0.088).

### Fewer alcohol-induced USVs upon nutritional ketosis

**Figure 5:**
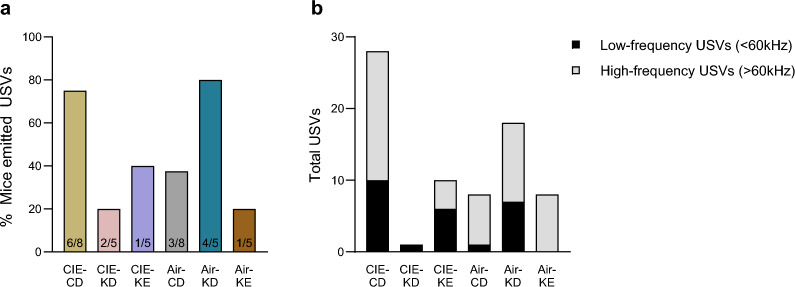
Fewer alcohol-induced USVs upon nutritional ketosis. The % of mice that emitted USVs during the 1st hour of alcohol OSA (**a**) and the total number of USVs emitted (**b**), subdivided in low- and high-frequency (< or > 60 Hz). Numbers at the bottom of each column represent number of mice that emitted USVs over total number of mice tested (**a**). Ultrasonic vocalizations (USVs).

Due to practical limitations, only 5–8 mice per group were tested in two PR OSA sessions. 6 of 8 mice in the CIE-CD group emitted USVs (28 USVs in total), 3/8 air-CD (8 USVs), 1/5 CIE-KD (1 USV), 2/5 CIE-KE (10 USVs), 4/5 air-KD (18 USVs), and 1/5 air-KE (8 USVs (Fig. 5a). The total number of USVs emitted was categorized in high-frequency > 60 Hz and low-frequency < 60 Hz (Fig. 5b).

Only 2 CIE-CD (2 USVs), 2 CIE-KD (2 USVs), 1 CIE-KE (11 USVs) and 1 air-KE (1 USV) mice emitted USVs during the tail suspension tests, whereas neither air-CD nor air-KD emitted any USVs.

### KE may modulate serotoninergic but not noradrenergic alcohol-induced signaling impairment

**Figure 6:**
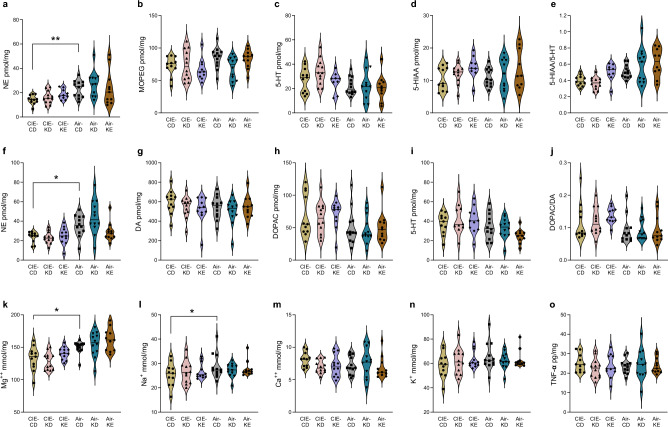
Neurochemical alterations following alcohol exposure and nutritional ketosis. The levels of NE (**a**), MOPEG (**b**), 5-HT (**c**), 5-HIAA (**d**), and 5-HIAA/5-HT ratio (**e**) in the hippocampus. The levels of NE (**f**), DA (**g**), DOPAC (**h**), 5-HT (**i**), and DOPAC/DA ratio (**j**) in the striatum. The concentration of cations Mg^++^ (**k**), Na^+^ (**l**), Ca^++^ (**m**) and K^+^ (**n**), in the cerebellum. The brain levels of TNF-α (**o**). Data are shown as individual subjects, horizontal line in violin plots represents group means. n = 10–12. *p < 0.05 and **p < 0.01.

CIE-CD mice displayed decreased NE levels in the hippocampus and striatum (Fig. 6a,f). In the hippocampus, two-way ANOVA revealed significant overall effects of alcohol for NE, 5-HT, 5-HIAA/5-HT ratio and a trend for 3-Methoxy-4-hydroxyphenylglycol (MOPEG) (F_1,59_ = 21.1, *p* < 0.0001, F_1,59_ = 8.5, *p* = 0.005, F_1,58_ = 22.9, *p* < 0.0001 and F_1,60_ = 3.4, *p* = 0.07 respectively), and of diet for HIAA-5-HT ratio (F_2,58_ = 5.5, *p* = 0.007). Post hoc analyses revealed significantly lower levels of NE and a trend for lower 5-HIAA/5-HT ratio in the hippocampus of CIE-CD mice compared to air-CD mice (*p* = 0.003 and *p* = 0.1; Fig. 6a), with the latter showing a trend towards amelioration by KE (*p* = 0.1), but no differences for MOPEG, 5-HT, and 5-HIAA (all *p* > 0.1; Fig. 6b-e). In the striatum, two-way ANOVA revealed significant overall effects of alcohol for NE, DOPAC, 5-HT, and DOPAC-DA ratio (F_1,61_ = 24.4, *p* < 0.0001, F_1,61_ = 9.4, *p* = 0.003, F_1,59_ = 14, *p* = 0.0004 and F_1,61_ = 9.5, *p* = 0.003 respectively), and a significant alcohol-diet interaction for NE (F_2,61_ = 3.9, *p* = 0.03). Post hoc analyses revealed statistically significantly lower levels of NE in the striatum of CIE-CD mice compared to air-CD mice (*p* = 0.03; Fig. 6f), but no differences for DA, DOPAC, 5-HT, and DOPAC/DA ratio (all *p* > 0.5; Fig. 6g-j). No statistically significant differences were found in the hippocampus and striatum between air-control groups (all *p* > 0.1).

### Alcohol exposure induced cation imbalances in the cerebellum

Cations levels were reduced in the cerebellum of CIE-CD mice (Fig. 6k-n). Two-way ANOVA revealed statistically significant overall alcohol effects for Mg^++^ and Na^+^ (F_1,62_ = 35.2, *p* < 0.0001 and F_1,62_ = 5.3, *p* = 0.02 respectively). A significant diet effect for Mg^++^ (F_2,62_ = 3.2, *p* = 0.047) seemed to reflect increased levels for KE, but there were no effects of diet for Ca^++^ and K^+^. Post hoc analyses indicated that CIE-CD mice had significantly lower Mg^++^ (*p* = 0.04; Fig. 6k) and Na^+^ (*p* = 0.03; Fig. 6l) levels compared to air-CD, as well as a trend for lower K^+^ levels (*p* = 0.08; Fig. 6n). No differences were detected for either Ca^++^ (Fig. 6m) or between air-control mice (all *p* > 0.3).

### Brain TNF-α levels were not affected by either alcohol exposure or nutritional ketosis

Two-way ANOVA revealed no effect of alcohol, diet nor alcohol -diet interaction for TNF-α levels (F_1,59_ = 0.6, *p* = 0.4, F_2,59_ = 0.5, *p* = 0.6 and F_2,59_ = 1.9, *p* = 0.16 respectively; Fig. 6o).

### Principal component analysis (PCA) revealed differential clustering between each diet pair

**Figure 7:**
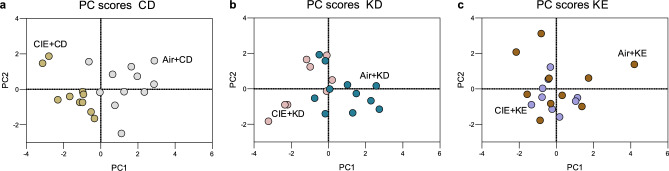
PCA reveals differential clustering between each diet pair. Principal component scores for the CD (**a**), KD (**b**), and the KE (**c**) pairs. Data are shown as individual principal component scores for each subject. n = 8–11. Principal component (PC).

Each diet pair was analyzed separately (Fig. 7a-c). PCA for the CD pair highlighted a clear separation between the CIE and air exposure groups, with factor 1 representing 44.9% and factor 2 accounting for 19.1% of the data variance. PCA for the KD pair revealed a distinctly smaller division between the CIE- and air-exposed groups. Indeed, factor 1 accounted for 38.3%, factor 2 for 20.1%, and factor 3 for 18.2% of the data variance, consistent with the KD reversing some of the alterations induced by alcohol. PCA for the KE pair revealed an even more unified cluster, with factor 1 accounting for 29.4%, factor 2 for 23.1%, factor 3 for 16.2%, and factor 4 for 14.9% of the data variance. This is consistent with the KE also reversing some of the alterations induced by alcohol.

### No changes in alcohol self-administration

CIE-CD mice did not display any significant difference in the number of reinforcers taken and the number of active pokes compared to air-CD mice and CIE-KD and CIE-KE mice did not differ from the CIE-CD mice. No statistically significant differences were found in either parameter between air-control groups (all *p* > 0.6; Supplementary Fig. 1a, b).

### Neither alcohol nor nutritional ketosis affected nociception

CIE-CD mice did not display any statistically significant alteration in the latency to first escape compared to air-CD mice (*p* = 0.093) and neither CIE-KD (*p* = 0.8) nor CIE-KE mice (*p* = 0.17) differed from the CIE-CD mice (Supplementary Fig. 1c). No statistically significant differences (all *p* > 0.5) were found in the latency to first escape between air-control groups.

The results are summarized in Table 1.

**Table 1:** Summary of behavioral and biochemical effects KD and KE in alcohol-exposed mice.

	KD	KE
Light/dark	(+)	Ø
Tail suspension	+	(+)
Saccharin preference	+	+
NE	Ø	Ø
5-HIAA/5-HT	Ø	(+)
Mg^++^	Ø	Ø
Na^+^	Ø	Ø

Only assays where there was a significant effect of alcohol are listed. + denotes a significant improvement compared to CIE-CD mice; (+) denotes a trend or mixed results; Ø indicates no effect.

## Discussion

Long-lasting alcohol withdrawal symptoms are characterized by complex and subtle neuronal mechanisms and are key contributors to relapse. In the present study we assessed the efficacy of nutritional ketosis, in the form of KD and KE supplementation, in ameliorating long-lasting alcohol withdrawal symptoms. We recently reported that the C57BL/6JRj mouse strain is more suitable than the C3H/HeNRj strain to study long-term alcohol withdrawal, in female mice, and we established a battery of behavioral and biochemical tests, which we implemented in the present report^30^. Both KD and KE reduced the severity of several alcohol withdrawal symptoms and they also showed a trend towards improving neurochemical alterations caused by chronic alcohol exposure, followed by long-term abstinence and re-exposure. Indeed, both KD and KE ameliorated long-lasting depressive-like symptoms, whereas only KD partially improved early anxiety-like disturbances.

Depression- and anhedonia-like disturbances are among the most challenging and long-lasting alcohol withdrawal symptoms^40–42^. We detected clear depression- and anhedonia-like symptoms following chronic intermittent alcohol exposure for almost the entire duration of abstinence, which is consistent with previous reports^30,43–45^. In the present study, we show for the first time that both KD and KE provided lasting and effective reduction of these symptoms in female mice. The effects are consistent with anti-depressive-like effects of nutritional ketosis in other rodents models used to induce depressive-like behaviors^46,47^. We hypothesize that these anti-depressants effects are driven by restoration of neurotransmitters homeostasis, specifically GABA and glutamate^18,48^.

CIE-CD mice displayed early anxiety-like symptoms in the light–dark test, but no long-lasting effects the same test or in the zero maze. We previously showed that KD rescued these acute anxiety-like disturbances, however here we only had a slight tendency for improvement^17^. This could be due to the decreased distance travelled by both CIE-CD and CIE-KE mice compared to air-CD mice on withdrawal day 1, which was further evident for the KE since air-KE mice travelled significantly less than air-CD on both withdrawal days. This could be a confounder, but, even though hypolocomotion has been often described during early alcohol withdrawal, the most consistent and pharmacologically validated parameter for the light/dark test was the number of light/dark transitions^49,50^. Long-lasting anxiety-like symptoms have proven challenging to measure in mice, with only one study describing them for up to three weeks following a 30-day (but not 14-day) binge drinking model in male C57BL/6J mice, in the elevated plus maze^44,51^. For the zero maze, lack of statistical power may have obscured mild effects in the present study since only two cohorts were tested. Both on withdrawal days 19^th^ and 26^th^, CIE-CD showed marginally reduced time spent in the open arms compared to CIE-KD and air-CD. It cannot be excluded that different timepoints and larger sample size could identify prolonged anxiety-like disturbance and effects of KD. Anxiolytic effects of nutritional ketosis have been recently described by Ari and colleagues^52^, who showed reduced anxiety-like symptoms upon KE administration in rats. Moreover, another recent study reported a dose-dependent anxiolytic effect of KE in female rats, with ketone levels in the range of 3 mM ameliorating anxiety-like symptoms in the light/dark test, while ketones within 4–5 mM, did not induce any change^53^.

A previous study reported the association between anticipatory 50 kHz USVs and the escalation of alcohol intake in dependent rats^38^. Following on our previous report, we found that a higher percentage of CIE-CD mice emitted USVs during OSA compared to air-CD, and they also emitted more USVs in total^30^. Interestingly, only one CIE-KD mouse and 2 CIE-KE mice emitted USVs. This might indicate that increased USVs emissions during alcohol withdrawal could reflect a general distress, which seems to be alleviated by nutritional ketosis. A recent study showed that a KD restored a neurodevelopmental communication deficit associated with decreased USVs in a rat model of intractable infantile spasm syndrome^54^. Very few mice emitted USVs during the tail suspension test, but this could be due to the short testing time.

CIE-CD mice did not escalate alcohol OSA and CIE-KD and CIE-KE mice did not display any difference in the present study. Similarly, CIE-CD mice did not show any signs of hyperalgesia. We recently reported that alcohol-exposed C57BL/6JRj mice did not escalate alcohol OSA using the same protocol, but they decreased their alcohol OSA more slowly compared to the control mice and they showed hyperalgesia^30^. This is in contrast to higher self-administered alcohol in vapor-exposed male rats relative to air-control, which was decreased by a history of KD^18^. As previously discussed, the long PR experimental sessions used could have masked the alcohol OSA escalation and produced high variability. It is possible that alcohol intake (more apparent using a fixed-ratio schedule of reinforcement), but not "motivation" or "seeking" is decreased by KD, consistent with a report showing that KD-treated mice responded for alcohol rewards but did not drink them^55^. It has been previously reported that male mice escalated their OSA following chronic alcohol exposure including using exposure by vapor inhalation and intragastric infusion^56,57^. Only one study has shown increased alcohol OSA in female mice, which however were not previously exposed to alcohol^58^.

Anti-inflammatory effects of KD have been previously described in a variety of preclinical rodent assays^22,27,46,59^. Conversely, alcohol has been shown to increase neuroinflammation^60,61^, and we therefore wanted to test whether ketosis ameliorated alcohol-induced neuroinflammation as part of its protective mechanism. We and others previously reported higher TNF-α central levels following alcohol exposure in mice^30,61^. In this study however, we did not detect a similar effect. However, a clinical trial found that switching from an American standard diet to an isocaloric KD increased glucose, total and low density lipoprotein cholesterol, and peripheral inflammation markers^62^. Thus, further investigations of different sample types and at multiple timepoints are needed to elucidate any potential anti- or pro-inflammatory effects of nutritional ketosis.

There is extensive evidence indicating alterations of dopaminergic, noradrenergic, and serotonergic signaling following alcohol exposure in both rodents and humans^63–67^. We did find lower NE levels in the hippocampus and in the striatum of CIE-CD mice compared to air-CD mice, consistent with a previous study in the frontal cortex and hippocampus of adult female rats^68^. The effects of alcohol on noradrenergic signaling are complex and dependent on timing, dose and route of administration but its dysregulation is a main factor of the pathophysiology of AUD^65,69^. We also detected marginally lower 5-HIAA/5-HT ratio in the hippocampus of CIE-CD mice compared to air-CD mice, which was moderately rescued by KE. The 5-HIAA/5-HT ratio is used as an estimate of serotoninergic activity and can provide useful information about the catabolic rate but has also important limitations. We found no other significant alterations in the concentrations of MOPEG, DOPAC, DA, 5-HT, 5-HIAA, and DOPAC/DA ratios in either the striatum or hippocampus of CIE-CD mice compared to air-CD mice. In exploratory studies, we previously described moderate cation imbalances in the cerebellum of alcohol-exposed mice^30^. Here, we found lower Mg^++^ and Na^+^ levels after CIE, similar to what has been reported in peripheral clinical samples^30,70,71^ .

Both KD and KE yielded sustained elevated blood βHB levels, reaching nutritional ketosis, i.e., blood ketone levels above 0.5 mM. KE produced higher βHB levels than KD after the first week and throughout the testing period. This was however in contrast with our previous study where KD rather than KE reached blood ketone levels up to 5 mM^17^. Sex and vendor differences as well as timing of blood collection could explain these divergences. Nutritional ketosis, achieved by either KD or KE supplementation, can elicit several metabolic effects such as lower plasma glucose levels and lower bodyweight gains in both rats and humans^15,19,62,72^. However, nutritional ketosis induced variable total and LDL cholesterol levels in both rodents and humans during physiological conditions^19,62,72–74^. The report by Clarke and colleagues^19^ is the only one investigating both sexes in rats showing a slight cholesterol increase after KE administration (30% calories from a ketone monoester). Meidenbauer and colleagues^74^ also showed increased cholesterol as well as triglycerides following KD ad libitum in male mice, but the amount of fat in the KD was 70%. Kemper and colleagues instead^73^, reported that KE supplementation (30% calories from the βHB ester) lowered plasma total cholesterol in male rats and mevalonate, a liver cholesterol synthesis biomarker, in humans. In the present study, we showed for the first time that not only KD but also KE reduced plasma glucose levels and KE also reduced weight gain at specific timepoints in female mice during alcohol exposure^73^ and withdrawal. There were differences in glucose levels over time, specifically for the CIE-KD mice. This could be related to changing conditions over time, such as alcohol exposure (first and last timepoint). Contrary to some previous studies in rodents and humans during physiological conditions, we found lower total cholesterol levels following KD administration, independently of alcohol exposure, and no changes in triglycerides^19,62^. The KE supplementation did not affect cholesterol or triglycerides levels. It has been hypothesized that substituting carbohydrate calories with either KD or KE could reduce the availability of acetyl-CoA for lipogenesis^73^. Together, our findings are consistent with good tolerability and low risk of deleterious effects of KD, with possibly higher risk of weight loss for the KE.

We conducted three separate PCA for each diet pair to better visualize and summarize the multiple dimensions associated with alcohol exposure and nutritional ketosis. There was a clear separation only between the alcohol and air exposed mice fed CD, whereas the PCA for the KD and KE pairs revealed more similar behavioral and biochemical patterns, suggesting that both KD and KE reversed at least some the alterations induced by alcohol. However, the air-KE had a different pattern compared to air-CD and air-KD mice, which could indicate a potential for aversive side effects.

We are aware of some limitations of this study. First, only female mice were included. Second, the timing of start of the experimental diets might be important for the outcome, and effects may not generalize to shorter diet exposure before onset of withdrawal. We previously described reduced alcohol withdrawal induced by nutritional ketosis when the diets were administered from the beginning of alcohol exposure and also when started at abstinence. For the latter, only KE was effective, likely due to a faster onset of blood βHB elevation relative to KD^17^. In the present study, diets were introduced in the last week of alcohol exposure, to mimic a possible clinical design. Lastly, the biochemical endpoints were measured at a single timepoint, after the final alcohol re-exposure. The time needed to process all samples caused the first mice to be terminated 2 h after the last alcohol exposure, and the last, 4 h, which may have introduced variability in the results.

In conclusion, our findings show that the beneficial effects of nutritional ketosis in ameliorating acute alcohol withdrawal symptoms in mice are also evident for long-term symptoms. This could have a positive impact in preventing relapse in AUD individuals. In addition, some of the biochemical endpoints measured were also partially ameliorated by nutritional ketosis after re-exposure. Overall, compared to KE, KD seemed to better relieve alcohol withdrawal symptoms, despite producing lower βHB levels. This indicates that high ketone levels alone are not necessary or sufficient to produce better effects, rather it seems that providing fatty acids to fuel the entire metabolic pathway produces multifactorial beneficial effects. Thus, the underlying mechanisms are yet to be fully identified. Notably, the ketogenic manipulations alone did not have any effect on behavioral or biochemical measures other than reduced cholesterol levels and reducing the distance travelled at a specific timepoint, demonstrating their safety and tolerability^24,25,75^. Our results also extend previous findings from male to female rodents^16–18^. These results further support promising beneficial effects of nutritional ketosis for the treatment of alcohol withdrawal symptoms. Given the apparent differences between the KD and KE effects, further studies are needed to better understand the underlying mechanisms of action and refine ketosis-based treatments.

### Supplementary Information

Supplementary Information.

## Data Availability

The raw data supporting the conclusions of this article will be made available by request to the corresponding author, without undue reservation.
