# Supplementary material for: Nutritional ketosis as treatment for alcohol withdrawal symptoms in female C57BL/6J mice
Source: Sci Rep. 2024 Mar 1;14:5092. doi: 10.1038/s41598-024-55310-3 (PMC10907582; doi:10.1038/s41598-024-55310-3)
Supplement: Supplementary file 1 — Supplementary Information. [file 41598_2024_55310_MOESM1_ESM.pdf]

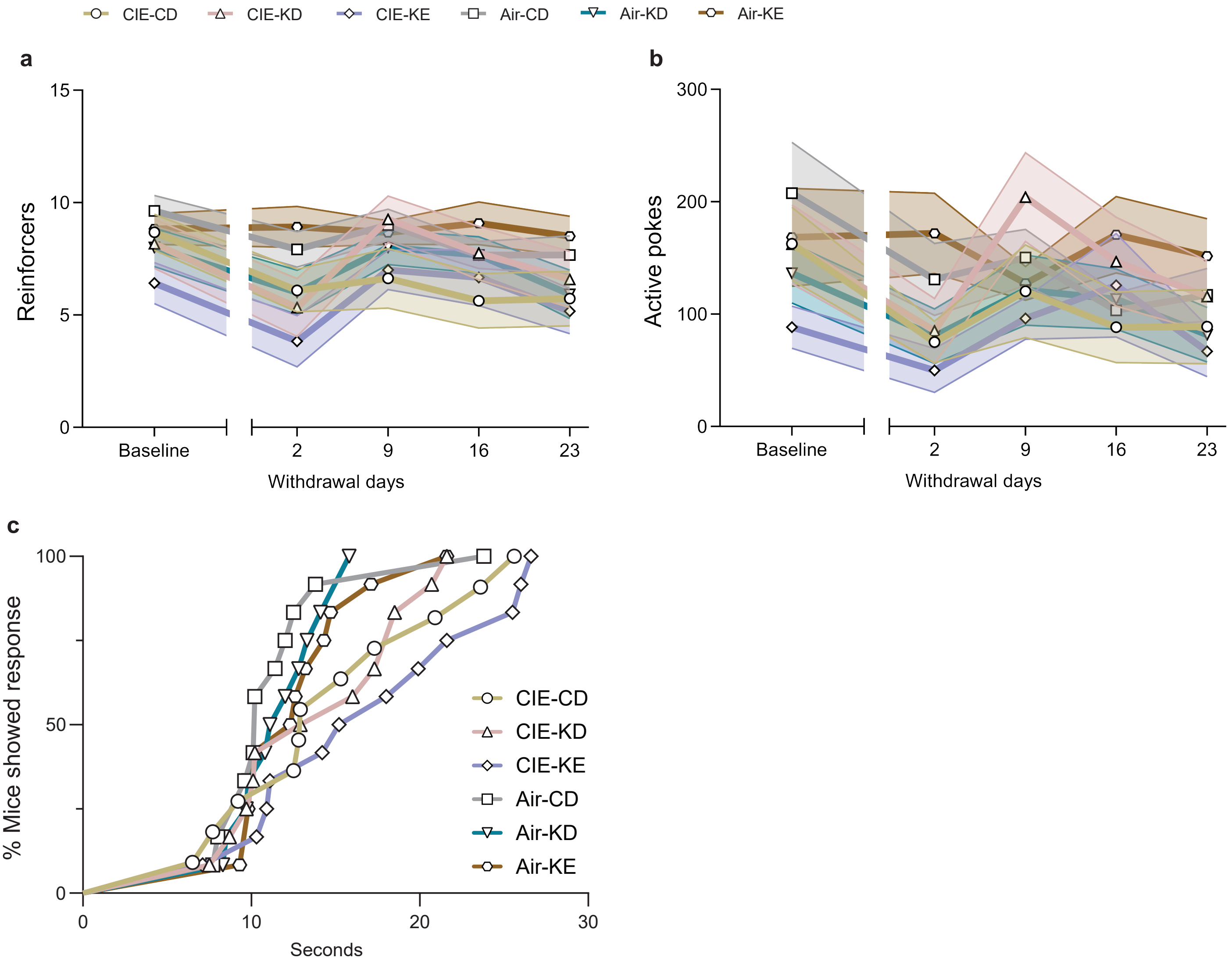

Supplementary Figure 1.

No effects of alcohol withdrawal or nutritional ketosis on alcohol OSA and nociception.
Number of reinforcers taken and of active nose pokes (a and b) during the six progressive ratio (PR) test sessions, where the baseline is the average of the first two PR sessions before the start of the experimental diets, and the following four PR sessions were performed with the experimental diets. Latencies in seconds to nociceptive response in mice tested on a 52°C hot-plate at withdrawal day 1 (c). Data are shown as group means, with the shaded area representing ± SEMs (a and b). Data are shown as the cumulative % of mice showing a response as a function of time (c). Note the truncated abscissa in panel a and b. n = 11-12.
